# The tRNA-Dependent Biosynthesis of Modified Cyclic Dipeptides

**DOI:** 10.3390/ijms150814610

**Published:** 2014-08-21

**Authors:** Tobias W. Giessen, Mohamed A. Marahiel

**Affiliations:** Department of Chemistry/Biochemistry and LOEWE Center for Synthetic Microbiology (SYNMIKRO), Philipps-University Marburg, Hans-Meerwein-Strasse-4, 35032 Marburg, Germany

**Keywords:** tRNA-dependent biosynthesis, cyclic dipeptides, cyclodipeptide synthases, CDPS (cyclodipeptide synthases), diketopiperazines, nocazines, combinatorial biosynthesis

## Abstract

In recent years it has become apparent that aminoacyl-tRNAs are not only crucial components involved in protein biosynthesis, but are also used as substrates and amino acid donors in a variety of other important cellular processes, ranging from bacterial cell wall biosynthesis and lipid modification to protein turnover and secondary metabolite assembly. In this review, we focus on tRNA-dependent biosynthetic pathways that generate modified cyclic dipeptides (CDPs). The essential peptide bond-forming catalysts responsible for the initial generation of a CDP-scaffold are referred to as cyclodipeptide synthases (CDPSs) and use loaded tRNAs as their substrates. After initially discussing the phylogenetic distribution and organization of CDPS gene clusters, we will focus on structural and catalytic properties of CDPSs before turning to two recently characterized CDPS-dependent pathways that assemble modified CDPs. Finally, possible applications of CDPSs in the rational design of structural diversity using combinatorial biosynthesis will be discussed before concluding with a short outlook.

## 1. Introduction

Aminoacyl-tRNA synthetases (aaRSs) represent an ancient and ubiquitous tRNA-utilizing enzyme family that possesses the unique catalytic capability of specifically attaching amino acids to the correct tRNA-adaptors needed during the translational process [1]. Thus, they represent the true decoders of the genetic code [2]. Due to their central function in the expression of genetic information they have been extensively studied over the past fifty years or so, resulting in important structural, enzymological and physiological insights related to various cellular processes. However, only relatively recently could it be shown that some atypical aaRSs or aaRS homologs can also act as peptide ligases themselves involved in secondary metabolite biosynthesis as well as the posttranslational modification of proteins. In fact, a clear progression from conventional aaRSs to paralogous enzymes possessing ligase activity can be proposed based on recent phylogenetic studies [3]. Some examples include dedicated aaRS paralogs that provide aminoacyl-tRNAs for specific pathways, e.g., lipid aminoacylation [4,5], δ-aminolevulinic acid biosynthesis [6,7], and valinamycin biosynthesis [8]. Examples where aaRS homologs act as ligases include the formation of mycothiol [9], as well as the aminoacylation of a conserved lysine residue in elongation factor P, which is essential for cell survival [10,11]. Here, we focus on another recently identified homologous enzyme family referred to as cyclodipeptide synthases that shows the largest sequential and functional divergence among all known aaRS homologs.

## 2. Cyclodipeptide Synthases

**Figure 1:**
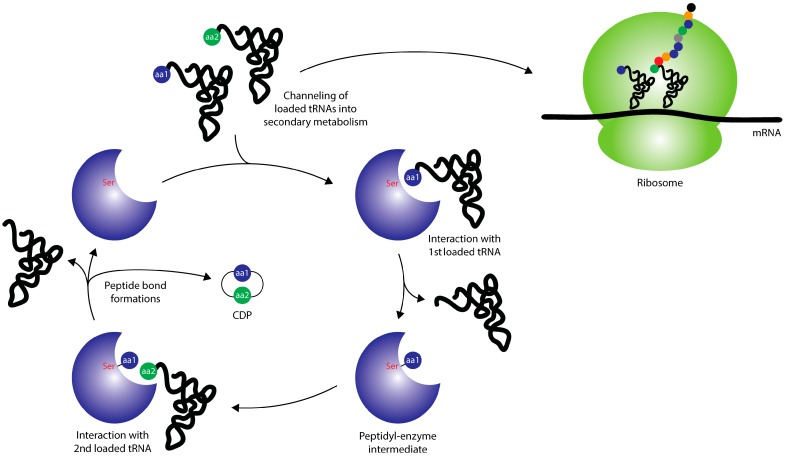
Overview of the general action of CDPSs (cyclodipeptide synthases) and their connection to primary metabolism. CDPSs hijack aminoacyl-tRNAs and employ them in the formation of CDPs (cyclic dipeptides), thus diverting the flow of loaded tRNAs away from the ribosomal machinery. Adapted from [31] with permission of The Royal Society of Chemistry, copyright 2012.

**Figure 2:**
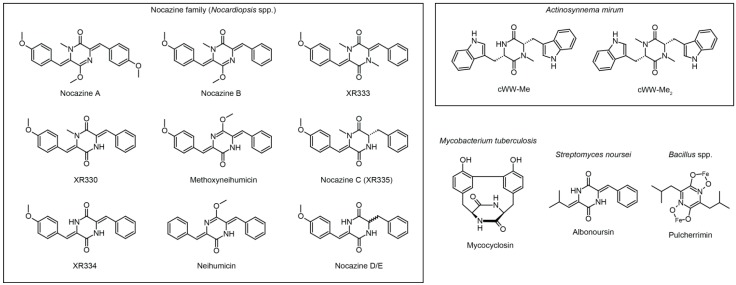
Structures of modified CDPs known to be produced in tRNA-dependent CDPS pathways.

Owing to the diverse and interesting bioactivities observed for many naturally occurring cyclic dipeptides, ranging from antibacterial [12,13,14] to immunosuppressive [15], the conformationally rigid and three-dimensionally defined cyclic dipeptide (CDP)-ring has long been recognized as a privileged scaffold for the generation of synthetic or semi-synthetic bioactive compounds [16]. On the other hand, very little is known about the *in vivo* functions of modified CDPs. It has been suggested that they might be involved in different biochemical communication phenomena [17,18,19,20] due to their small size and their ability to diffuse through membranes reminiscent of many lactone-based quorum sensing autoinducers [21,22,23]. Regarding the biosynthetic origins of CDP-containing natural products, it was traditionally thought that nonribosomal peptide synthetases (NRPSs) are responsible for their assembly, either through dedicated biosynthetic pathways or through premature release of dipeptidyl intermediates during the chain elongation process [24,25,26]. However, the discovery of AlbC, an enzyme able to specifically form CDPs using loaded tRNAs as substrates, revealed a second dedicated route for the production of cyclic dipeptides [27,28]. To date, eleven homologs of AlbC could be characterized and many more have been identified using PSI-BLAST searches [29,30,31]. Together they constitute the family of tRNA-dependent cyclodipeptide synthases. Through the use of aminoacylated tRNAs as substrates, CDPSs actively divert activated amino acids from the ribosomal machinery and represent a direct link between primary and secondary metabolism (Figure 1). So far, only few CDPS-dependent biosynthetic pathways have been elucidated. Those are the biosynthetic routs leading to the antibiotics albonoursin [27,28] and mycocyclosin [27,28,32,33,34,35], the siderochrome pulcherrimin [36,37,38], the nocazine family [30], and, finally, methylated ditryptophan CDPs (Figure 2) [29].

### 2.1. Distribution and Organization of CDPS (Cyclodipeptide Synthase) Gene Clusters

**Figure 3:**
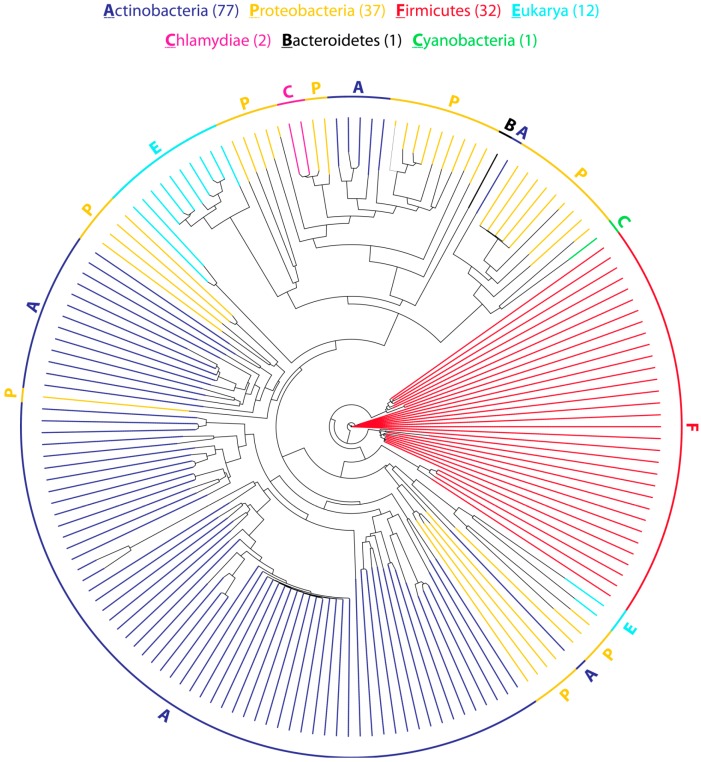
Phylogenetic tree representing the evolutionary relationship of 162 putative CDPSs. The tree is based on a multiple sequence alignment generated using Clustal Omega [39,40]. The optimal tree was inferred using the Neighbor-Joining method. Evolutionary distances are in units of the number of amino acid substitutions per site.

As of May 2014, more than 150 putative CDPS genes could be identified using BLAST searches (Figure 3). They are distributed among six bacterial phyla (Actinobacteria, Bacteroidetes, Chlamydiae, Cyanobacteria, Firmicutes, and Proteobacteria), as well as four eukaryotic phyla (Ascomycota, Annelida, Ciliophora, and Cnidaria). The vast majority of CDPSs is present in bacteria with only twelve putative CDPS genes found in eukaryotic organisms. Thus far, no archaeal CDPS genes could be identified. However, this does not necessarily reflect the real distribution of CDPSs because the availability of sequenced genomes is heavily skewed towards plant, animal and human pathogens.

**Figure 4:**
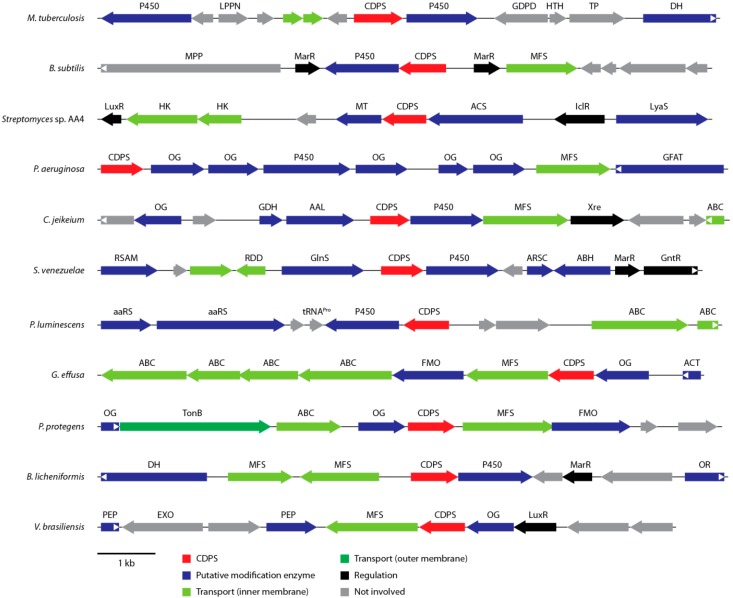
Overview of an exemplary selection of putative CDPS gene clusters. Modification enzymes, regulators and transporters are indicated by the color code defined at the bottom of the figure. Abbreviations: AAL: d-Ala-d-Ala ligase; aaRS: aminoacyl-tRNA synthetase; ABH: α/β-hydrolase; ACS: acyl-CoA synthetase; ACT: acyl-CoA transferase; DH: dehydrogenase; FMO: flavin monooxygenase; GlnS: glutamine synthase; MT: methyltransferase; OG: α-ketoglutarate/Fe^2+^-dependent oxygenase; OR: oxidoreductase; P450: cytochrome P450; PEP: peptidase; PLP: PLP-dependent oxidase; RSAM: radical SAM enzyme.

CDPSs from bacteria of the same phylum generally cluster together, although some proteobacterial CDPSs can be found in actinobacterial clades and vice versa. Only four genes (two in Chlamydiae, one in Bacteroidetes and one in Cyanobacteria) that are not found in Actinobacteria, Proteobacteria, or Firmicutes could be identified with all of them being quite closely related to proteobacterial CDPSs. This could indicate that many CDPS genes may have undergone horizontal gene transfer. In addition, some organisms (e.g., *Streptomyces cattleya* and *Rickettsiella grylli*) encode multiple non-paralogous CDPS genes that are only distantly related and may be able to produce different sets of cyclic dipeptides. In general, at least one putative tailoring enzyme is found in the direct genetic surroundings of each bacterial CDPS gene, indicating that the initially assembled CDP is almost always further modified [31]. The associated modification enzymes belong to a variety of different enzyme families, among them many oxidoreductases, hydrolases, ligases, and transferases (Figure 4). By far, the most common class of putative tailoring enzymes found near CDPS genes are various kinds of oxidases including at least seven distinct types of cytochrome P450s, five different types of α-ketoglutarate/Fe^2+^-dependent oxygenases, as well as three distinct flavin-containing monooxygenases. In addition to oxidoreductases, different *O*-, *N*-, and *C*-methyltransferases, α/β-hydrolases, acyl-CoA transferases, as well as peptide ligases have been identified in putative CDPS gene clusters hinting at a diverse set of modifications that can be introduced into CDPS-dependent cyclic dipeptides [31]. In most CDPS clusters, different kinds of transcription factors belonging to the LuxR and MarR families among others can be observed. They are usually involved in regulating various processes in response to environmental stimuli like resistance to toxic chemicals and antibiotics, the expression of virulence factors and the adaptation to oxidative stress, which may hint at the biological function of CDPS-dependent modified CDPs [41,42]. In addition, different membrane transporters are often found in close proximity to CDPS genes either involved in transport processes through the outer membrane of Gram-negative bacteria (TonB-dependent receptors) or the inner membrane (ATP-binding cassette (ABC)-type transporters, sodium solute symporters, and major facilitator transporters) [43]. All the genetic associations mentioned above suggest that CDPS gene clusters produce highly modified cyclic dipeptides in response to environmental cues, which are then exported to either the periplasm or extracellular space.

### 2.2. Structural Aspects and Enzymology

**Figure 5:**
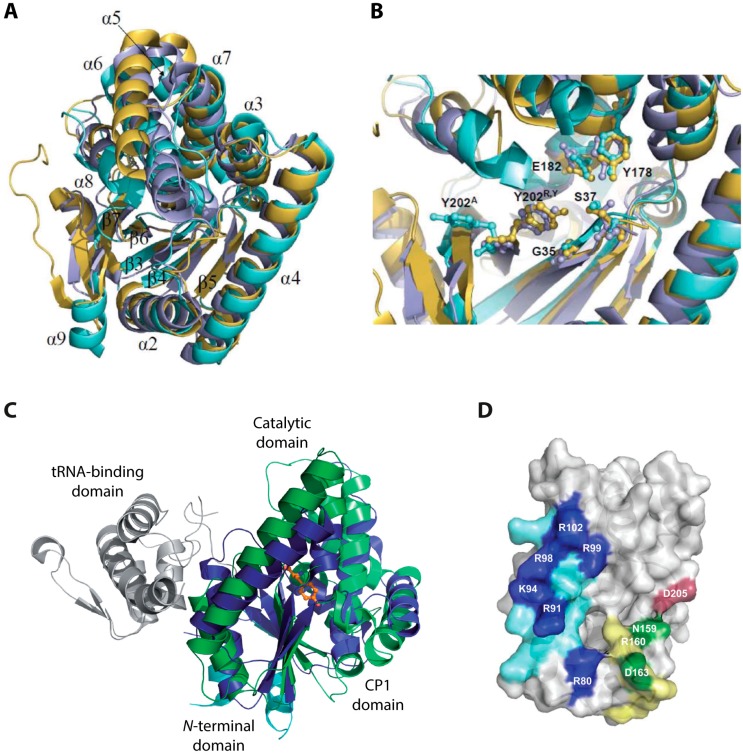
(**A**) Superposition of the AlbC (cyan, PDB (Protein Data Bank) ID: 3OQV), Rv2275 (yellow, PDB ID: 2X9Q) and YvmC-Blic (purple, PDB ID: 3OQH) structures. The numbering of β-strands and α-helices corresponds to AlbC; (**B**) Superposition of the conserved residues inside the active site pocket. The residues are shown in ball and stick style. AlbC numbering is used; (**C**) Comparison of the structures of AlbC and TyrRS^Tyr^ from *M. jannaschii* in complex with l-Tyr (PDB ID: 1JLU). The enzymes are shown in cartoon style, l-Tyr is shown in ball and stick style in orange. The Rossmann-fold and CP1 domain of TyrRS^Tyr^ are colored in dark and light blue, respectively. The corresponding domains of AlbC are shown in dark and light green. The tRNA-binding domain is colored grey; and (**D**) Regions in AlbC involved in the interaction with tRNA substrates (PDB ID: 3OQV). Basic residues located in helix α4 are colored in blue. Residues shown in dark blue have been shown to strongly influence the interaction with the first aminoacyl-tRNA substrate. Loop α6-α7 is colored in yellow and D205 in loop β6-α8 in red. Green and red residues have been shown to influence binding of the second tRNA substrate. (**A**,**B**) Adapted from [31] with permission of The Royal Society of Chemistry, copyright 2012; (**C**) Adapted from [44] with permission from Oxford University Press, copyright 2011; and (**D**) Adapted from [52] with permission from Oxford University Press, copyright 2014.

Many insights that shed light on various aspects of CDPS enzymology have been gained from the three crystal structures (AlbC, *S. noursei*; YvmC, *B. licheniformis*; Rv2275, *M. tuberculosis*) that could be determined so far [35,36,44]. Although sharing only moderate sequence identity, the structures superimpose very well (Figure 5A) with rms deviations of only 2.27 Å (for Rv2275 *vs.* AlbC over 192 C_α_ atoms), 2.2 Å (for Rv2275 *vs.* YvmC over 211 C_α_ atoms), and 2.46 Å (for AlbC *vs.* YvmC over 180 C_α_ atoms). Regarding their oligomeric state, AlbC and YvmC could be shown to be active monomers in solution, while Rv2275 showed a homodimeric quaternary structure in gel filtration experiments. Each CDPS monomer adopts a compact α/β-fold consisting of five parallel β-strands surrounded by α-helices (Rossmann-fold domain: β3-β5, α2 and α4) [45]. Particularly the Rossmann-fold domain as well as strands β6 and β7 superimpose well in all three CDPSs. A surface accessible pocket that contains active-site residues important for substrate selection and catalysis is present in all structures. Five of the seven almost universally conserved residues in CDPSs are located within this pocket with four of them superimposing very well in all three structures with the exception of Tyr202 (AlbC numbering, Figure 5B).

Besides the similarities discussed above, the following differences could be observed in the solved crystal structures: a structured *N*-terminal region in Rv2275 that is not observed in AlbC and YvmC, a *C*-terminal region that is present in the AlbC structure but not in Rv2275 and YvmC and large deviations in loops β3-α2, α6-α7 (not observed in Rv2275), and β6-α8 that are likely involved in substrate binding. The three CDPS structures revealed a high structural similarity with the catalytic domain of class-Ic aaRSs, especially TyrRSs and TrpRSs [35,36,44]. CDPSs are particularly close in structure to the archaeal TyrRSs from *Archaeglobus fulgidus* and *Methanococcus jannaschii* and TrpRS from the eukaryote *Entamoeba histolytica* [35,36,44,46,47,48,49,50,51]. In addition to the very well conserved Rossmann-fold domain, TyrRSs/TrpRSs and CDPSs also contain a helical connective polypeptide 1 (CP1) subdomain (Figure 5C). Again, a few obvious differences between aaRSs and CDPSs exist: class-Ic aaRSs are generally homodimers in solution while CDPSs act as monomers (the previously mentioned homodimeric oligomerization state for Rv2275 is very likely not necessary for catalysis) [36,44], the conserved and required ATP binding motive present in aaRSs is not present in CDPSs reflecting the fact that they do not need ATP for catalysis and finally, CDPSs do not possess a distinct tRNA-binding domain but rather contain a large patch of positively charged residues located in helix α4 important for the binding of aminoacyl-tRNA substrates. All those observed differences provide CDPSs with their unique catalytic capabilities having basically reversed the role of aminoacyl-tRNAs in their catalytic cycle compared to their ancestral homologs. In contrast to aaRSs that yield loaded tRNAs as their reaction products, CDPSs use them as substrates to carry out a ligation reaction employing a completely different set of active site residues for catalysis.

The catalytic cycle of CDPSs is governed by the fact that two loaded tRNAs are sequentially employed for cyclic dipeptide formation. The cycle is initiated by the binding of the first aminoacyl-substrate, likely involving ionic interactions between the negatively-charged ribose-phosphate tRNA backbone and the abovementioned positively charged helix α4 [35,36,44]. Enzyme and tRNA mutagenesis studies have shown that the aminoacyl moiety of the first substrate binds in the previously mentioned solvent exposed pocket and that CDPS substrate specificity for the first loaded tRNA is mainly directed at the aminoacyl moiety. In addition, the tRNA acceptor stem sequence, especially the N_1_-N_73_ pair, and to a smaller degree the identity of the second aminoacyl moiety have been shown to be important for efficient binding of the second substrate. It could be shown that AlbC is not able to use all Leu-tRNA^Leu^ isoacceptors as second substrates with the G_1_-C_73_ pair of the acceptor stem being essential for efficient recognition of this second substrate [52]. Turning now to the specificity determinants of the CDPSs themselves, the particular amino acids lining the active site within the surface accessible pocket have been shown to exert a strong influence on which first aminoacyl substrate can be efficiently bound. It was even possible to rationally change the specificity of AlbC from mainly cFL to mainly cYL by introducing a single L200N point mutation in the active site, underlining the importance of those residues in the selection of the first substrate [44]. By mutating residues in the AlbC loops α6-α7 and β6-α8 it was recently shown that those loops do affect the specificity for particular Leu-tRNA^Leu^ isoacceptors, strongly indicating that they are critical for the interaction with the second aminoacyl-tRNA substrate (Figure 5D) [52]. Those results clearly show that AlbC possesses two distinct binding sites for its two substrates. Interestingly, two mutations in these loops, D205A and D163A, yielded AlbC variants with higher catalytic activity, possibly reflecting an evolutionary trade-off between improving enzyme specificity while losing some catalytic efficiency. Another explanation for this phenomenon could be that those two residues may reduce the affinity of cognate aminoacyl-tRNAs (tRNA^Phe^ and tRNA^Leu^) to AlbC on purpose, ensuring that the major fractions of those loaded tRNAs are available for ribosomal protein synthesis.

**Figure 6:**
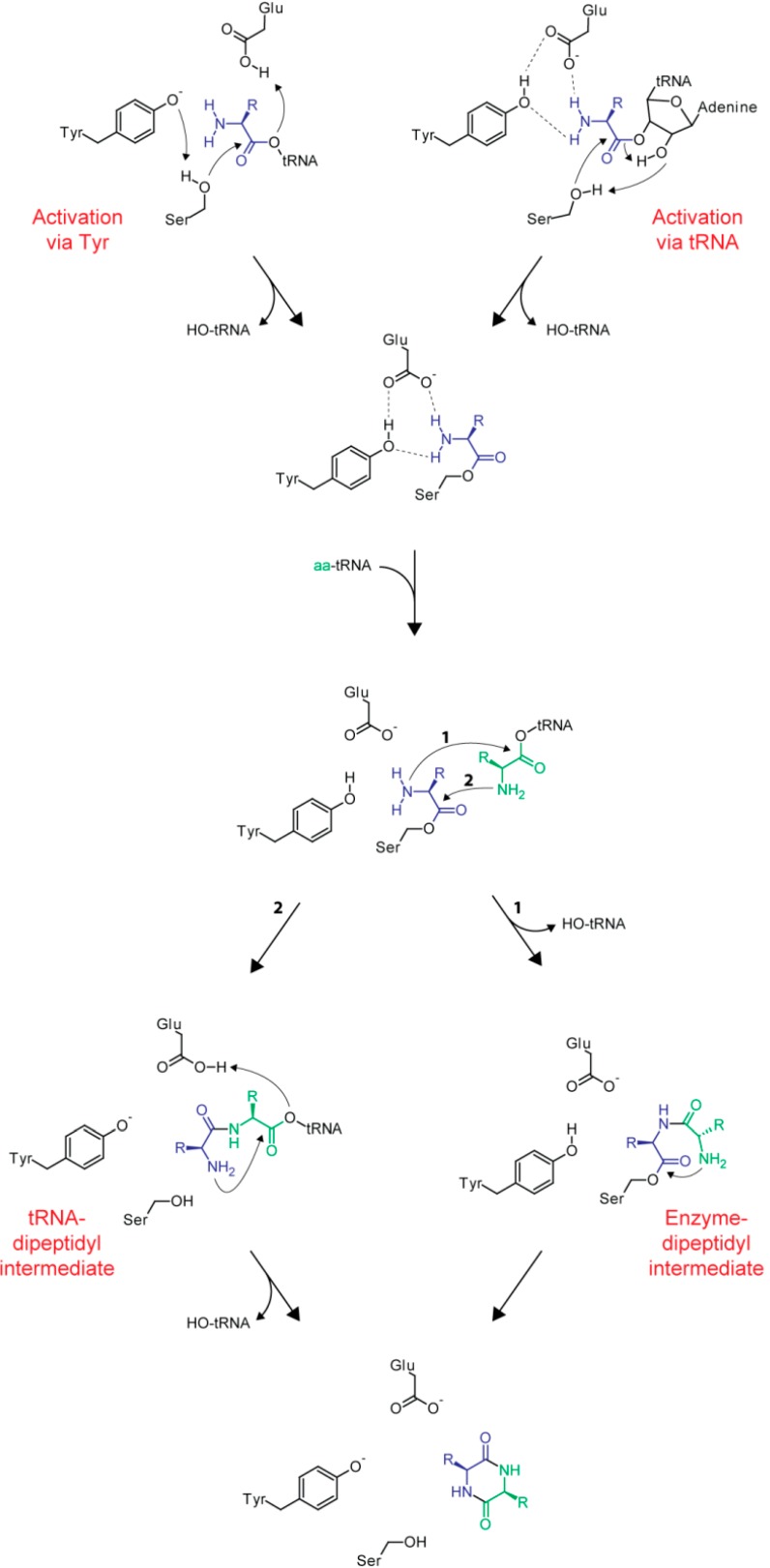
Proposed ping-pong mechanism of CDPS catalysis. Activation of the active site nucleophile (Ser) can be accomplished through interaction with a conserved tyrosine (**left**) or via a proton-shuttling mechanism involving the tRNA substrate (**right**). After the covalently bound aminoacyl-enzyme intermediate has been formed either an enzyme-dipeptidyl (**right**) or tRNA-dipeptidyl (**left**) intermediate is generated. Through attack of the α-amino group of the second aminoacyl moiety, the CDP is formed and released from the enzyme.

The catalytic mechanism of CDPSs can be described by a sequential ping-pong model (Figure 6). After specific recognition of the first substrate, the first aminoacyl group is transferred to a conserved serine residue located inside the solvent exposed pocket. Tandem mass spectrometry, as well as radioactive labeling experiments, were used to unequivocally show the formation of this peptidyl-enzyme intermediate [44,53]. Two different mechanisms have been proposed for the initial activation of the catalytic serine residue needed for subsequent attack onto the reactive oxoester of the first loaded tRNA. Firstly, the side chain hydroxyl group of a conserved tyrosine could engage in hydrogen bonding with the active site nucleophile resulting in its increased nucleophilicity as observed in a mutational study with Rv2275 [35]. On the other hand, a concerted proton-shuttling mechanism that involves the two vicinal hydroxyl groups of nucleotide A76 of the tRNA molecule has been proposed mimicking nucleophile activation in the ribosomal peptidyl transferase and FemX (*W. viridescens*) [54,55]. The structures obtained for Rv2275 and YvmC indicate that access to the active site located in the relatively deep pocket discussed above is quite restricted. It was proposed that those structures may represent closed enzyme states with aminoacyl-tRNA binding needed to initially convert CDPSs to their active open state. While the exact influence of tRNA-binding remains unclear, it seems obvious that extensive remodeling of the loops restricting access to the active site is needed to allow binding of the first aminoacyl moiety. 

In addition to the actual active site nucleophile, a second highly conserved residue inside the surface accessible pocket (E182, AlbC numbering) exists that seems to be important for the coordination of the α-amino group of the substrate aminoacyl moiety. Together with another conserved residue (Y178) this glutamate plays a critical role in the correct positioning of the aminoacyl group for subsequent nucleophilic attack by the catalytic serine residue [31]. After initial formation of an aminoacyl-enzyme intermediate the covalent substrate-enzyme complex would then react with the second loaded tRNA to form either a dipeptidyl-enzyme or dipeptidyl-tRNA intermediate. Via intramolecular cyclization formation of the second peptide bond would at the same time release the readily assembled CDP from the enzyme. Thus far, none of the two mentioned dipeptidyl-intermediates could be detected.

## 3. tRNA-Dependent CDPS Pathways—Two Recent Examples

Recently, two formerly unknown biosynthetic systems have been identified and characterized that are able to produce modified CDP natural products in a tRNA-dependent fashion [30]. In the following sections the lessons learned and insights gained from those studies will be presented and placed in the wider context of natural product biosynthesis.

### 3.1. The Nocazine Family

**Figure 7:**
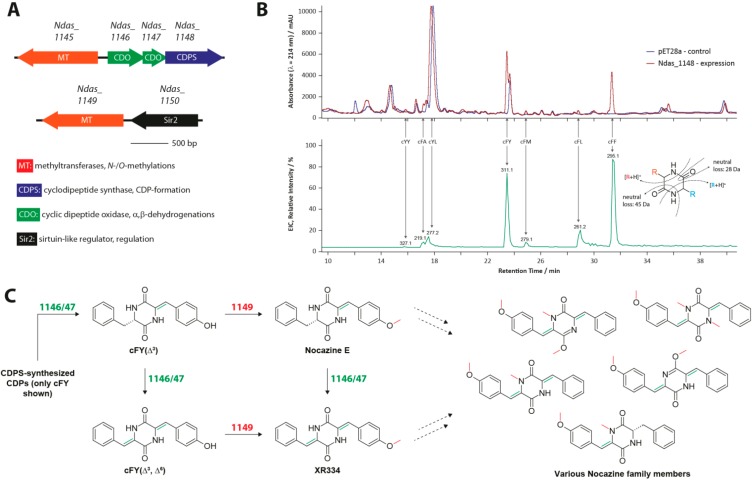
(**A**) Biosynthetic gene cluster of the nocazine family; (**B**) Liquid chromatography-mass spectrometry (LCMS) chromatogram showing the products of the CDPS Ndas_1148; and (**C**) Overview of the nocazine biosynthetic pathway [29]. Investigated CDP-modifications are shown in red and green.

In two recent studies, four formerly unknown highly modified CDPs, together with three known and highly similar compounds, could be isolated from *Nocardiopsis dassonvillei* and *Nocardiopsis alba* [56,57]. The authors were focused on their isolation and structure elucidation and were not concerned about their biosynthetic origins. Following up on those initial studies, we started our investigation based on the hypothesis that those compounds, from now on referred to as nocazines, are synthesized via a CDPS-dependent route. Initially, a bioinformatic analysis of the *N. dassonvillei* genome was conducted resulting in the identification of the first putative nocazine gene cluster (Figure 7A). It was subsequently shown that this gene cluster is responsible for the production of a whole range of related modified CDPs constituting the nocazine family.

All members of this family share the following structural features: they all consist of two aromatic amino acids, at least one of which is oxidized to its α,β-dehydrogenated form and in addition, at least one *O*- or *N*-methylation is present either in a side chain or the central six-membered CDP-ring itself. The biosynthetic pathways for nocazine E and XR334 could be completely reconstructed starting with the initial generation of two CDPs catalyzed by the tRNA-dependent CDPS Ndas_1148. This CDPS possesses a formerly unknown product profile forming cFY and cFF as its main products along with at least five additional CDPs (Figure 7B). Although being quite promiscuous, Ndas_1148 shows a preference for Tyr/Phe-loaded tRNAs. This behavior closely resembles the substrate specificities observed for most other characterized CDPSs [31]. Further modification of the CDP-scaffold is then achieved through the combined and combinatorial action of a cyclic dipeptide oxidase (CDO) and two distinct SAM-dependent *O*-/*N*-methyltransferases [58,59]. The CDO Ndas_1146/1147 is a large oligomeric protein complex consisting of two small subunits capable of introducing α,β-dehydrogenations into a wide variety of different CDPs suggesting that it recognizes the heterocyclic CDP-core and not any particular amino acid constituents of a given CDP. The next step in nocazine biosynthesis is then carried out by the *O*-methyltransferase Ndas_1149 by methylating the side chain hydroxyl group of tyrosine. Finally the *O*-/*N*-methyltransferase Ndas_1145 introduces *O*- or *N*-methylations in the CDP-ring. The biosynthetic pathway discussed above represents the first CDPS pathway that contains more than one tailoring enzyme directly involved in CDP modification. Interestingly, all enzymes that participate in the nocazine pathway show relaxed substrate specificities enabling the generation of an array of structurally related compounds by a single gene cluster encoding only four small genes (Figure 7C). This exemplifies a common strategy often used in nature to produce structural diversity, namely the differential tailoring of a core scaffold through the combinatorial use of various promiscuous modification enzymes. In general, small molecule diversity facilitates the evolution of completely new biological functions and in addition enables the fine-tuning of already existing ones [60,61].

### 3.2. Methylated Tryptophan-Containing Cyclic Dipeptides 

**Figure 8:**
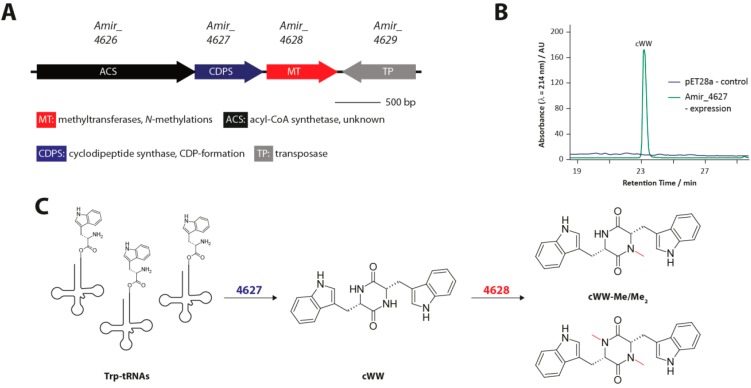
(**A**) Biosynthetic gene cluster for methylated ditryptophan CDPs; (**B**) LCMS chromatogram showing the single product of the CDPS Amir_4627; and (**C**) Overview of the biosynthetic pathway leading to singly and doubly methylated ditryptophan CDPs in *A. mirum*. Adapted from [29] with permission from American Chemical Society, copyright 2013. Investigated CDO (cyclic dipeptide oxidase)-modifications are shown in red.

Tryptophan-containing CDPs are among the most prevalent cyclic dipeptides found in nature [16]. Many have been isolated from a wide range of microbial organisms, particularly different fungal *Aspergillus* and *Penicillium* species. In contrast, comparatively few CDPs that contain a tryptophan residue have been isolated from bacteria so far. In a recent study [29], we used a genome mining approach to identify a new CDPS gene cluster in the actinobacterium *Actinosynnema mirum* (Figure 8A). In the course of our investigation we characterized the newly discovered CDPS Amir_4627, as well as the *N*-methyltransferase Amir_4628. The two end products of this tRNA-dependent natural product pathway are singly and doubly methylated ditryptophan CDPs. The two most unusual features of this pathway are the very high substrate specificity of Amir_4627 which generates only cWW (Figure 8B) and the identity of the resulting CDP because all previously identified bacterial CDPSs generate products containing different combinations of the four amino acids phenylalanine, tyrosine, methionine and leucine [31]. From a structural perspective CDPSs are particularly similar to TyrRSs and TrpRSs indicating that those subclasses of aaRSs may have been their evolutionary ancestors. Amir_4627 is more closely related to Nvec-CDPS2, a eukaryotic CDPS also able to synthesize tryptophan-containing CDPs, than to all other characterized CDPSs, possibly suggesting that they derive from a TrpRS precursor, while most other CDPSs may derive from a TyrRS precursor [53]. The unusually high specificity of Amir_4627 probably stems from its very specific interaction with the second tryptophanyl-tRNA substrate. In light of a recently published study investigating CDPS specificity determinants, loop α6-α7 of Amir_4627 together with the particular stem loop sequence of *E. coli* tRNA^Trp^ could be responsible for the observed high specificity [52]. The initially generated cWW is successively *N*-methylated at the CDP-ring nitrogens by the promiscuous *N*-methyltransferase Amir_4628 (Figure 8C), which has been shown to methylate various other CDPs containing large and/or aromatic amino acids. A large number of bacterial and fungal enzymes are known that modify tryptophan-containing CDPs, which makes the identification of a potent catalyst for cWW-formation an interesting first step for the generation of artificially modified cyclic dipeptides. Additional possibilities for combinatorial *in vivo*, as well as chemoenzymatic approaches to rationally generate small molecule structural diversity using the CDP-scaffold will be further discussed in the following section.

## 4. Rational Design of Structural Diversity Using CDPSs

Two general approaches to rationally modify the structure of peptide natural products exist. Firstly, the peptide backbone itself can be altered by changing the identity, number or connectivity of the constitutive amino acids. Secondly, an already assembled peptide scaffold can be differently decorated by tailoring enzymes that introduce certain chemical modifications resulting in a structurally and often functionally altered natural product [61].

CDPs are naturally quite limited regarding the modulation of their peptide backbone as they consist of only two building blocks arranged with a predefined connectivity. It follows that besides the ligation of additional peptidic building blocks to side chain functional groups present in the CDP, only the alteration of monomer identity does represent a feasible diversification approach. CDPS-derived CDPs possess an additional limitation regarding their structure, imposed by the intrinsic catalytic properties of the CDPS family, since the use of aminoacyl-tRNAs as substrates dictates that only the 20–22 proteinogenic amino acids do represent valid building blocks for the assembly of CDPs. As discussed above, CDPS-specificity mainly depends on the identity of the aminoacyl moiety bound to the tRNA, although recent results indicate that the first base pair in the acceptor stem (N_1_-N_73_) can have a marked influence on the recognition of the second aminoacyl-tRNA substrate, as well as product formation and thus CDPS specificity [52]. This implies that diversification of the CDP-scaffold can be achieved by changing either the building block carried by a certain tRNA or the sequence of a tRNA that is specific for a particular amino acid. Standard mutagenesis methods or* in vitro* transcription can be employed to introduce small specific sequence changes into tRNAs (e.g., N_73_ mutants) which generally do not influence the overall structure of the tRNA and do not hinder aminoacylation [62]. On the other hand, to change the amino acid loaded onto a specific tRNA, two approaches could be envisioned. In both cases techniques originally developed for the introduction of non-proteinogenic amino acids into proteins could be used to generate CDPs containing unnatural or non-standard monomers.

**Figure 9:**
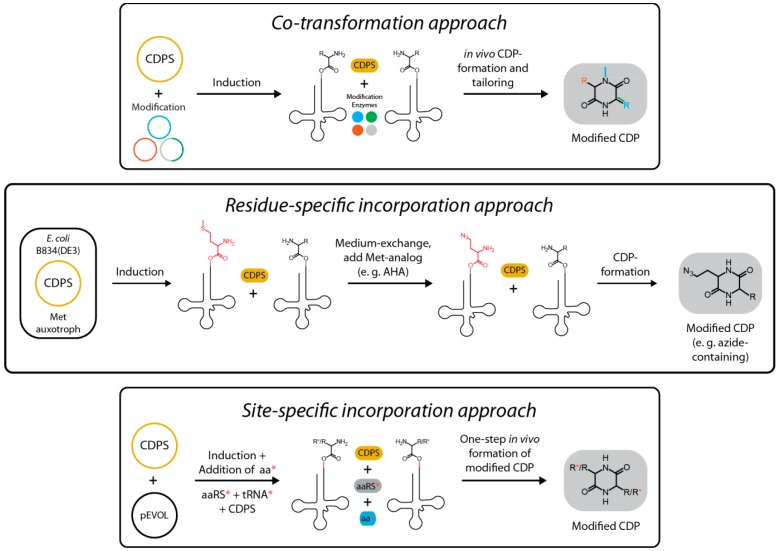
Different approaches for the rational generation of structural diversity using CDPSs. Shown are the co-transformation approach employing various modification enzymes to introduce new structural features into the CDP-scaffold, the residue-specific incorporation approach which relies on the use of amino acid analogs and auxotrophs and the site-specific incorporation approach where an orthogonal aaRS/tRNA pair is used to incorporate non-proteinogenic amino acids into CDPs.

The first strategy is referred to as residue-specific incorporation and yields a globally modified proteome containing a desired non-canonical building block at all positions normally occupied by a certain proteinogenic amino acid [63]. This is achieved by omitting a natural amino acid of choice in the growth medium while supplying a non-canonical analog combined with the use of auxotrophs as expression hosts to obtain high-level replacement. While no genetic manipulation is required in this approach, the range of non-canonical building blocks that can be recognized and processed by the natural translation machinery (aaRSs and the ribosome) is relatively limited. To use this strategy for the production of CDPs containing unnatural amino acids, the non-canonical building blocks do not need to be recognized by the ribosome, but only by the respective aaRSs. In fact, such a scenario would be beneficial for the production of CDPs due to an increase in the amount of usable aminoacyl-tRNA substrates for a given CDPS (Figure 9). The second strategy that could be used to incorporate non-proteinogenic monomers into CDPS-derived CDPs is referred to as site-specific incorporation [64,65]. This method was initially developed to introduce point mutations into proteins with minimal perturbation of a given protein structure. First, an orthogonal tRNA/aaRS pair that was evolved to activate a specific unnatural building block is introduced into an expression host. Then, the respective monomer is added to the growth medium. This building block has to be able to diffuse into the cell resulting in its incorporation into proteins. This method relies on the amber suppression technology that enables the site-specific incorporation of a specific non-canonical amino acid into a protein dependent on the presence of the amber codon, which can be easily introduced at any position in any protein using standard genetic engineering techniques. In analogy to residue-specific incorporation, the unnaturally loaded tRNA will not only be used by the ribosome but will at the same time serve as a substrate for a CDPS generating CDPs that contain unnatural building blocks (Figure 9).

The second general strategy to alter the structure of peptide natural products is the tailoring of an already assembled peptide scaffold by specific modification enzymes [61]. For the purpose of modifying CDPs, not only enzymes found in CPDS gene clusters may be considered, but, in addition, enzymes found in other biosynthetic pathways (e.g., NRPS pathways) known to use CDPs as substrates. This opens up the possibility of constructing artificial hybrid pathways for the production of highly modified CDPs using tailoring enzymes originating from various unrelated biosynthetic gene clusters (Figure 9). The small size of CDPS genes and the high yields of CDPS-derived CDPs together with an easy purification process make synthetic pathways based on CDPs synthesized by CDPSs ideal candidates for* in vivo* fermentation approaches aimed at the production of new modified cyclic dipeptides. Up until now, only five of the putative tailoring enzymes identified in CDPS-gene clusters have been studied, with most of them displaying a relatively broad substrate specificity which would make them useful for CDP diversification approaches. The various kinds of putative CDPS tailoring enzymes have already been discussed in Section 2.1 and will not be further discussed here. Instead, we will subsequently focus on CDP-modifying enzymes found in NRPS-gene clusters. In Table 1, a small selection of gene clusters involved in the production of NRPS-dependent tailored CDPs is shown. It is evident that a wide variety of different reactions can be catalyzed by modification enzymes found in NRPS-gene clusters and that various CDP-scaffolds can serve as valid substrates. It is noteworthy, that the majority of known NRPS-derived CDPs are produced by fungi, whereas comparably few bacterial CDP-producers are known. Interestingly, many fungi produce trypthophan-containing CDPs that are very often further modified by different prenyltransferases (PTs) [66,67]. In recent years, PTs that modify all positions of the indole ring in tryptophan-containing CDPs could be characterized and represent a possibly valuable repertoire of CDP-diversification catalysts (Table 2) [68]. Prenylated natural products often possess biological activities clearly distinct from their non-prenylated precursors, which makes PTs especially interesting for the construction of synthetic pathways for bioactive “unnatural” natural products. Considering that an enzyme capable of producing high amounts of cWW has recently been identified and characterized (Amir_4627), the rational or combinatorial generation of differently prenylated and further modified CDPS-derived CDPs is within reach.

**Table 1:** Exemplary selection of NRPS (nonribosomal peptide synthetases) pathways generating cyclic dipeptide (CDP)-containing natural products. Shown are the different modification enzymes found inside those gene clusters, their putative function as well as the substrate CDP-scaffold used by those enymes.

Biosynthetic Pathway	Modification Enzymes	Putative Function	CDP Substrate
Thaxtomin [24,69,70] (*Streptomyces scabies*)	TxtC	Hydroxylation	cWY
Brevianamide [71] (*Aspergillus fumigatus*)	Afu8g00240	Oxidative cyclization	cWP
	Afu8g00230	Oxidative cyclization	
	Afu8g00220	Hydroxylation	
	Afu8g00200	*O*-methylation	
	Afu8g00190	Hydroxylation	
Ergotamine [72] (*Claviceps purpurae*)	CpP4501	Hydroxylation	cFP
	CpCAT2	Hydroperoxidation	
	CpOX3	Oxidative cyclization	
Meleagrin [73] (*Penicillium chrysogenum*)	Pc21g15430	*C3*-reverse-prenylation	cWH
	Pc21g15440	*O*-methylation	
	Pc21g15450	Oxidative cyclization	
	Pc21g15460	*N*-hydroxylation	
	Pc21g15470	α,β-dehydrogenatioin	
Acetylazonalenin [74] (*Neosartorya fischeri*)	AnaPT	*C3*-reverse-prenylation	cWF
	AnaAT	*N*-acetylation	
Gliotoxin [75] (*Aspergillus fumigatus*)	GliC	Oxidation	cFS
	GliF	Oxidation	
	GliG	Sulfurization	
	GliI	Cyclopropane-formation	
	GliM	*O*-methylation	
	GliN	*N*-methylation	

**Table 2:** Overview of different prenyltransferases able to modify all viable positions in the indole side chain of tryptophan residues.

Organism	Enzyme Name	Reaction	Modification Position
*Aspergillus fumigatus*	FtmPT2 [76]	Prenylation	N1
*Aspergillus fumigatus*	FtmPT1 [77]	Prenylation	C2
*Neosartorya fischeri*	CdpC3PT [78]	Reverse Prenylation	C3
*Aspergillus fumigatus*	FgaPT2 [79]	Prenylation	C4
*Aspergillus clavatus*	5-DMATS [68]	Prenylation	C5
*Streptomyces* sp. SN-593	IptA [80]	Prenylation	C6
*Aspergillus oryzae*	CTrpPT [81]	Prenylation	C7

## 5. Conclusions and Outlook

In conclusion, the biosynthesis of modified cyclic dipeptides by CDPSs represents the so far most complex and widely distributed tRNA-dependent process reliant upon aaRS homologs found in secondary metabolism. Although much has been learned about CDPSs over the past ten years, many open questions still remain, including: what are the exact specificity determinants that determine if a given CDPS produces a whole set of CDPs or only a single compound? What new kinds of modifications can be introduced into CDPs by various tailoring enzymes? For what purpose and under which conditions are modified CDPs produced in nature? These are only some of the broader still inadequately answered questions regarding CDPSs. Finally, it will be interesting to see in which way CDPSs and their associated modification enzymes will be employed in synthetic biology approaches aimed at the production of valuable small molecules. In the end, as is often the case, possible applications will strongly depend on the vision and creativity of researchers and their ability to apply the fundamental insights gained in interesting and meaningful ways.
